# Multi-Band Enhanced Energy Harvesting from Dual Sources Using a Symmetrical Gradient Metamaterial Beam

**DOI:** 10.3390/s25237266

**Published:** 2025-11-28

**Authors:** Weiqiang Mo, Yubin Lin, Shiqing Huang, Dongqin Li, Rongfeng Deng, Fengshou Gu

**Affiliations:** 1 School of Industrial Automation, Beijing Institute of Technology, Zhuhai 519088, China; weiqiang.mo@hud.ac.uk (W.M.); shiqing.huang@hud.ac.uk (S.H.); dongqin.li@hud.ac.uk (D.L.); rongfeng.deng2@hud.ac.uk (R.D.); 2 Centre for Efficiency and Performance Engineering, University of Huddersfield, Huddersfield HD1 3DH, UK

**Keywords:** multi-band, energy harvesting, dual-source, rainbow trapping, metamaterial

## Abstract

Wireless sensors are vital for real-time condition monitoring of rotating machinery. Traditionally, these sensors depend on batteries, a solution that is neither eco-friendly nor cost-effective due to high maintenance. Vibration energy harvesting has emerged as a promising alternative for powering these sensors. Nevertheless, current energy harvesters commonly disregard high-frequency energy, which is weak but contains abundant condition-monitoring information. Moreover, the destructive interference between multiple vibration sources further attenuates the high-frequency energy. To address these limitations, this paper proposed a novel energy harvesting method based on a symmetrical gradient metamaterial beam (SGMB). The SGMB structure is designed to have multiple bands to enhance the high-frequency energy and diminish the destructive interference of flexural waves from two vibration sources. Multiple piezoelectric patches are integrated into SGMB to convert the dynamic stress into harvestable electrical power, enabling multi-band dual-source energy harvesting. Based on the rainbow trapping mechanism, the SGMB was first designed and optimized for desired frequency bands. Subsequently, the band characteristics and piezoelectric output performance were adjusted and validated through finite element simulation. Finally, experimental evaluations were conducted to validate the performance of the designed metamaterial. The results demonstrate that the SGMB provides multiple enhanced bands within the range from 1000 Hz to 3500 Hz and improves the energy harvesting efficiency by a factor over 100, which represents a breakthrough in developing self-powered and self-sensing wireless sensors.

## 1. Introduction

As rotating machinery becomes increasingly complex to achieve high integration, high speed, and intelligence, the acquisition of condition signals for monitoring and fault diagnosis has become more challenging, necessitating a greater number of sensors for comprehensive data transmission [1,2]. It means that sensing system can also be more complicated and have higher costs and lower reliability. In addition, extensive wiring is required for data transmission between the transducers, sensors and the terminal to monitor the system. Systems that use a mass of wires have other basic disadvantages, such as high cost, difficult installation, and unreliable communication [3,4]. To address these limitations, the reliable wireless sensors have emerged as the optimal solution [5]. Wireless sensor devices play a critical role in acquiring high-quality signals [6,7,8]. In addition, wireless sensor networks offer advantages such as low power consumption, high adaptability, and integrated intelligent features for machinery condition monitoring [9,10]. However, powering these wireless sensors poses a challenge due to application constraints. Dependence on batteries requires high maintenance and replacement, posing significant economic and environmental burdens [11,12,13]. An attractive alternative is vibration energy harvesting, to enable self-powered monitoring systems that ensure long-term and maintenance-free sensor operation. Furthermore, by analyzing the electrical signals, some self-powered sensors allow for the self-sensing of mechanical conditions [14].

Research on linear vibration energy harvesters shows that significant power can be output only when the ambient excitation frequency is close to or equal to the natural frequency of harvester [15,16,17]. This is a fundamental requirement for powering wireless sensors. Therefore, these vibration energy harvesters can only work in a very narrow low-frequency band, neglecting the collection of high-frequency energy [18,19]. This high-frequency energy, although weak, contains rich condition monitoring information, which facilitates the development of a self-powered and self-sensing device [20]. Therefore, widening the high-frequency operating bandwidth of vibration energy harvesters is crucial for boosting both the power supply and sensing capabilities of sensors.

Practical industrial machinery often contains multiple vibration sources, where the destructive interference of flexural waves between different sources greatly diminishes energy harvesting efficiency, while also corrupting the condition information embedded within those signals. A crucial step in multi-source energy harvesting is to mitigate destructive interference and even leverage the superposition of flexural waves to enhance vibrations [21,22]. Consequently, significant research efforts have been dedicated to investigating this key issue. Huang et al. developed a wave energy harvester capable of dual-frequency power capture, which efficiently harvests energy from wind waves at two distinct frequencies [23]. Palmer et al. utilized constructive interference to enhance conversion efficiency and boost the performance of their wave energy converter [24]. However, these approaches can only mitigate wave destructive interference at a single frequency or in a narrow frequency band, making them unsuitable for industrial machinery with multi-source, high-frequency broadband excitation characteristics. It is imperative to develop a multi-source, high-frequency vibration energy harvester to solve the energy supply issue for self-sensing devices in high-frequency environments, paving the way toward self-powered, self-sensing technologies.

In recent years, considerable research attention has been devoted to metamaterials, which are artificially engineered periodic composite structures possessing unique physical properties not found in natural materials [25,26]. The emergence of metamaterials has provided advanced pathways for piezoelectric vibration energy harvesting, motivating researchers to utilize these structures for multi-source broadband high-frequency energy harvesting [27,28,29,30]. For example, Chen et al. proposed an innovative rainbow piezoelectric energy harvesting approach utilizing a topological meta-device to achieve stable and efficient vibration energy capture [31]. Wang and colleagues investigated a graded metamaterial beam, numerically and experimentally validating the rainbow trapping effect on a gradually varied plastic beam fabricated through 3D printing [32]. By using piezoelectric patches with enhanced voltage signals from energy concentration as a replacement for conventional sensors, Mo et al. achieved machine fault detection with a significantly improved fault characteristic signal-to-noise ratio [33]. Chaplain et al. demonstrated the critical distinction between rainbow reflection and genuine rainbow trapping effects. By examining the interaction duration between slow zero-group-velocity waves and the resonator array, they revealed how this differentiation influences energy harvester design [34]. Significant progress has been made in energy harvesting to improve conversion efficiency and expand bandwidth in high frequencies. Nevertheless, emerging metamaterial-based enhancement methods remain susceptible to the destructive interference of flexural waves from multiple sources, posing a challenge to the realization of self-powered, self-sensing wireless devices.

This paper proposes a novel energy harvesting method based on a symmetrical gradient metamaterial beam (SGMB), which features multi-band enhanced energy concentration in the high-frequency range and suppression of destructive interference. With integrated piezoelectric patches to convert the dynamic stress into harvestable electrical power, the SGMB achieves dual-source vibration energy harvesting in high-frequency range. Furthermore, the development of the SGMB facilitates the use of lumped parameter models for beam segment modeling in preliminary system design. This process allows for the adjustment of piezoelectric output characteristics through finite element analysis (FEA) and the calibration of output voltage, enabling the design and optimization of a dual-source energy harvesting system for different frequency bands.

The remainder of this paper is organized as follows. Section 2 elaborates on the geometric design, piezoelectric output characteristics, optimization of matching resistance, and band structure analysis of the SGMB. Section 3 presents the results from frequency domain and piezoelectric output simulations. In Section 4, experimental calibration demonstrates the characteristics of spatial-frequency separation and energy concentration, experiments conducted on both shaker and rotor test rig validated the enhanced energy harvesting capability of the SGMB. Finally, Section 5 presents the key conclusions.

## 2. Symmetrical Gradient Metamaterial Structure Design

### 2.1. Construction of Metamaterial

**Figure 1:**
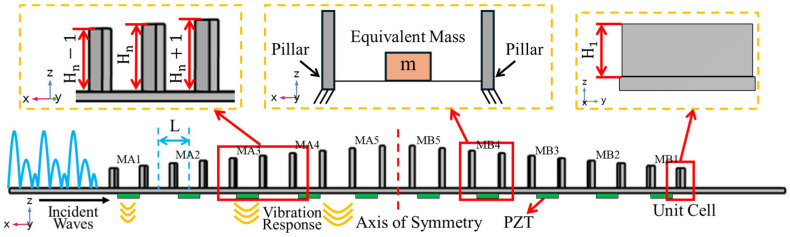
Schematic of symmetrical gradient metamaterial beam.

Figure 1 illustrates the proposed structure of SGMB, which consists of a base beam and 20 rectangular pillars. The length of the base beam is 310 mm, and the 20 pillars are symmetrically placed on both sides of the base beam, positioned vertically along its length. The rectangular pillars exhibit a symmetrical gradient in height, ascending from both ends toward the peak at the centre. In a single side, the height of the nth pillar is $H_{n}=H_{1}+\left(n-1\right)\;mm$, and $H_{1}=8\;mm$ is the height of the first pillar. The maximum height of the pillar is $H_{10}=17\;mm$. The structure features rectangular pillars and a base beam, both with a thickness of 2 mm. The pillars have a base length of 20 mm, matching the width of the beam, while the lattice constant is set at L = 12 mm in the gradient beam. For clarity of description, the gaps between pillars are designated in the order of MA1 to MA5 and MB1 to MB5, as shown in Figure 1. Ten Lead Zirconate Titanate (PZT) patches are glued on the bottom of each gap to convert mechanical vibration energy into electrical energy. Based on the sophisticated printing conditions, the material (polylactic acid) characteristic parameters of the prototype SGMB including Young’s modulus E=2.7 GPa, density $\rho =1250\;kg/m^{3}$, Poisson ratio v=0.34. The symmetrical pillars on the left and right sides form two sets of gradient structures, which converge and amplify the vibrational energy from both ends of the beam. As vibrational waves pass through the SGMB structure, they induce enlarged responses in each of the gaps.

### 2.2. Model for Piezoelectric Output

**Figure 2:**
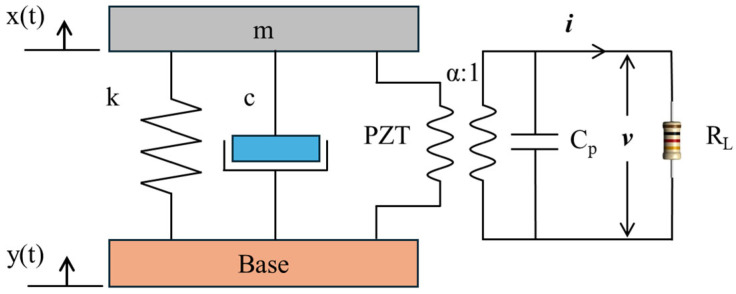
SGMB structure represented as an equivalent SDOF model.

Mounted at the base of each gap, these PZTs are capable of converting vibrational responses into amplified electrical signals over multiple frequency bands. Therefore, the output characteristics of PZTs significantly impact the sensing capability and energy harvesting performance of SGMB systems. The configuration of the metamaterial allows for each gap with a PZT to be conceptually modeled as the fixed beam. As shown in a magnified view of Figure 1, the first modal response of the beam can be analysed using an equivalent single-degree-of-freedom (SDOF) system comprising a mass, spring, and damper. As reported by Wang et al. [35], the voltage output v(t) generated by the displacement x(t) of each unit cell is predictable using the coupled model presented in Figure 2 according to Equations (1) and (2), respectively:

(1) $$m\ddot{x}\left(t\right)+c\dot{x}\left(t\right)+kx\left(t\right)+\alpha v\left(t\right)=c\dot{y}\left(t\right)+ky\left(t\right),$$

(2) $$\alpha \left[\dot{x}\left(t\right)-\dot{y}\left(t\right)\right]-C_{p}\dot{v}\left(t\right)=i\left(t\right)=\frac{v\left(t\right)}{R_{L}}.$$

Here, m signifies the lumped equivalent mass of the beam, c in turn denotes the damping coefficient, and k represents the equivalent stiffness. The displacement of the vibration is expressed as y(t) and x(t). Key parameters for the PZT are listed in Table 1, including the electromechanical factor α and the electrical capacitance $C_{p}$. Meanwhile, the electrical outputs across the load resistance are characterized by the current i(t) and voltage v(t). Assuming the base displacement takes the form $y\left(t\right)=Y_{0}e^{j\omega t}$, where $Y_{0}$ is the amplitude, ω is the frequency, and j is the imaginary unit, the subsequent feedback force exerted by the equivalent mass on the base can be expressed as:

(3) $$x\left(t\right)=\frac{j2\xi_{n}\omega_{n}\omega +\omega_{n}^{2}+\left(j\omega \alpha^{2}/m\left(j\omega C_{p}+\frac{1}{R_{L}}\right)\right)}{j2\xi_{n}\omega_{n}\omega +\omega_{n}^{2}-\omega^{2}+\left(j\omega \alpha^{2}/m\left(j\omega C_{p}+\frac{1}{R_{L}}\right)\right)}y\left(t\right),$$

where $\omega_{n}=\sqrt{k/m}$ and $2\xi_{n}=c/\sqrt{km}$. Substituting Equation (3) into Equation (2), the relationship between the stable output voltage across the load resistance and the base vibration can be established:

(4) $$v\left(t\right)=\frac{j\omega \alpha }{j\omega C_{p}+\frac{1}{R_{L}}}\times \frac{\omega^{2}}{j2\xi_{n}\omega_{n}\omega +\omega_{n}^{2}-\omega^{2}+\left(j\omega \alpha^{2}/m\left(j\omega C_{p}+\frac{1}{R_{L}}\right)\right)}y\left(t\right).\;$$

### 2.3. Optimal Resistance Analysis

**Figure 3:**
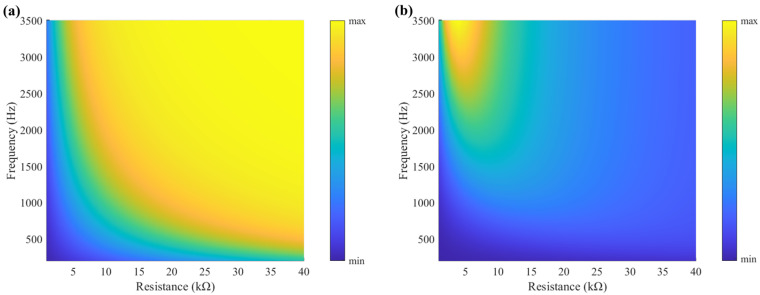
Electrical output of the PZT across different frequencies and resistances: (**a**) output voltage and (**b**) output power.

As shown in Figure 2, a simple circuit with a resistive load is connected directly to the output of the PZT. The value of this resistive load, which is critical to the output characteristics of the piezoelectric element, must match the internal impedance of the PZT to enable the extraction of a continuous and stable electrical signal from the resistive load. Figure 3a shows the output voltage predicted by Equation (4) across different frequencies and resistances. At a fixed frequency, once the resistance exceeds the matched value, the output voltage asymptotically approaches a maximum and remains stable. A higher excitation frequency requires a smaller matching resistance. Consequently, for signal acquisition, the chosen resistance must be sufficiently high to ensure a continuous and stable signal is obtained.

Furthermore, the voltage signals generated by the PZT contain substantial energy that can be recovered and utilized. For energy harvesting, a matched resistor plays a critical role in the circuit to maximize the output power. Based on Equation (4), this model enables the prediction of output power across varying frequencies and load resistances, as shown in Figure 3b. Because the internal impedance of PZT behaves as a capacitor, the optimal matching resistance for the circuit varies with the excitation frequency. Specifically, a higher frequency requires a lower matching resistance. At a fixed frequency, any deviation of the chosen resistance from the optimal matching value will significantly compromise the output efficiency of the piezoelectric element.

**Figure 4:**
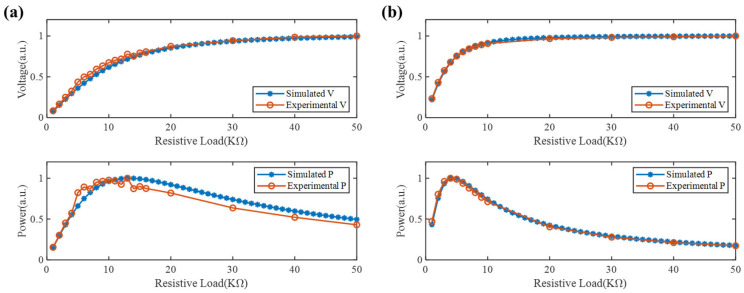
Comparison of simulated and experimental output characteristics: (**a**) output voltage and power for 1000 Hz and (**b**) output voltage and power for 3000 Hz.

To further verify the accuracy of Equation (4) in predicting the output characteristics of each PZT, the output voltage and output power of the piezoelectric unit in the SGMB prototype under different resistances were measured at excitation frequencies of 1000 Hz and 3000 Hz, respectively. Figure 4 illustrates the comparison of experimental results and simulated characteristics, where V refers to the output voltage, and P means the power. It can be observed that the output performance of the piezoelectric unit in the prototype shows good agreement with the predictions given by Equation (4), verifying that the piezoelectric unit has achieved the expected voltage and power output performance. Based on the analysis results, for voltage signal acquisition, a resistor greater than 40 kΩ should be used to ensure continuous and stable voltage signals. When performing energy harvesting within the excitation frequency range of 1000–3000 Hz, a resistor ranging from 4–13 kΩ should be selected to ensure high output power from the PZT.

### 2.4. Band Structure Analysis

**Figure 5:**
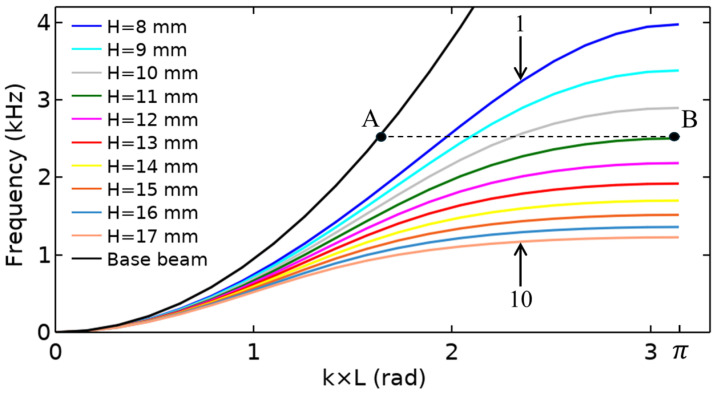
Band structure of SGMB.

The band characteristics of each single unit cell were examined through numerical simulations using a FEA model in COMSOL Multiphysics 5.6, revealing the spatial frequency selective properties of the SGMB. The implementation of Bloch-periodic boundary conditions was aligned with the wave propagation path. The research assigned free boundary conditions to all other surfaces of the unit cell. Since the two sets of pillars MA and MB are symmetrical, their spatial frequency selective properties are identical. Therefore, only 10 pillars in left side were selected to be analyzed. As depicted in Figure 5, the 10 gradually increasing unit cells are arranged in sequence from 1 to 10. Increasing the height of the rectangular pillars causes a downward shift in the first-order dispersion curves within the first Brillouin zone. The band structure of the SGMB is defined by this set of dispersion curves.

To elucidate how the group velocity of the incident wave varies with the height of the rectangular pillars and decreases to zero, it is necessary to explain the equation for group velocity:

(5) $$v_{g}=\frac{d\omega }{dk}=2\pi \frac{df}{dk},$$

which $v_{g}$ represents the group velocity, ω denotes the angular frequency, f indicates the frequency, and k stands for the wavenumber. As illustrated in Figure 5, the incident wave with constant frequency propagates in the base beam is shown by point A. When the wave propagates throughout the SGMB section, the gradually increasing pillar height implies a corresponding decrease in group velocity. The black horizontal dashed line is tangent to the green curve at point B, where the green curve represents the fourth pillar with a height of H=11 mm. The tangency with the horizontal line indicates that df/dk is zero, meaning the group velocity is zero. This point of tangency is referred to as the zero-group velocity (ZGV) point. Due to momentum remaining conserved, the ZGV point causes the flexural wave to cease forward propagation and experience reflection at its matching unit cell.

As a result, the specific frequency and ZGV point can be associated with each rectangular pillar. Each pillar in the SGMB acts as a reflection point for a wave of its corresponding frequency, causing the wave to be reflected. This phenomenon of spatial-frequency separation is known as the rainbow trapping effect. The reflection process causes the group velocity of the incident wave to diminish to zero, leading to a concentration of energy at the point of reflection. The superposition of incident and reflected waves significantly enhances the wave energy, resulting in the wave energy concentration effect of the rainbow trapping.

## 3. Performance Analysis of Metamaterial

The frequency associated with the ZGV point of each unit cell is defined as the cutoff frequency. Given the above analysis, the heights and cutoff frequencies of the ten rectangular pillars on one side of the SGMB are obtained, as listed in Table 2.

### 3.1. Frequency Domain Simulation

**Figure 6:**
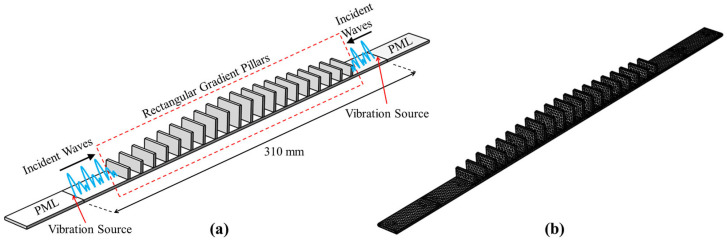
Model for frequency domain analysis: (**a**) schematic of the simulation setup and (**b**) mesh configuration.

To accurately investigate the frequency domain characteristics of the proposed metamaterial, a finite element analysis was conducted in the frequency domain. As shown in Figure 6a, two vibration sources for generating flexural waves are set at both ends of the frequency domain simulation model, with the acceleration specified as $0.1\;m/s^{2}$ and the cutoff frequency used as the sweep frequency for this analysis. After passing through the uniform section, the flexural waves propagate into the SGMB region. In the structural damping setting, the energy dissipation of the material was characterized by an isotropic loss factor, which was set to $\eta_{s}=0.01$. Furthermore, perfectly matched layers (PML) are applied at both ends of the gradient metamaterial beam to absorb all boundary reflections. Figure 6b shows the mesh configuration of the simulation model, which uses the pre-defined finer global element size to ensure the precision of the calculation results.

**Figure 7:**
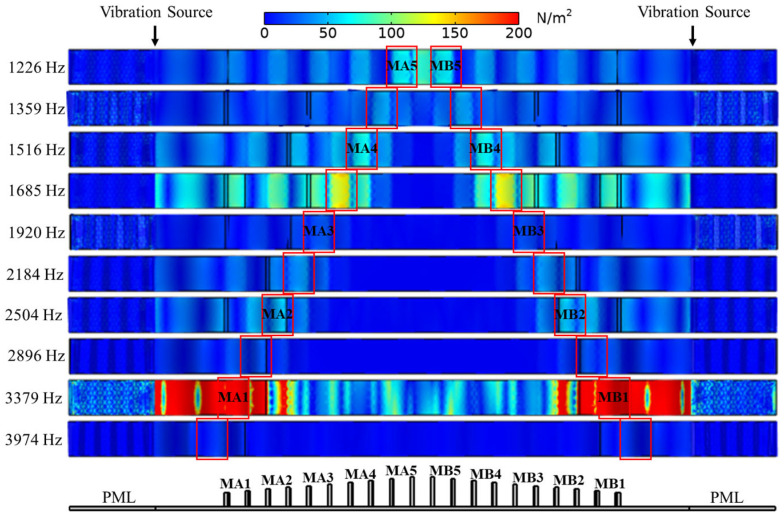
Spatial-frequency separation and energy concentration on SGMB.

The numerical results are shown in Figure 7, which displays the frequency responses of the SGMB at each cutoff frequency within consecutive gaps. The bottom surfaces of the corresponding gaps are labeled in the sequence of MA1–MA5 and MB1–MB5 in Figure 7. A more distinct frequency response is achieved by analyzing every other pillar gap. When incident waves of different frequencies travel through the SGMB, they encounter corresponding cutoff unit cells where propagation ceases and reflection occurs. The sharp stress decline observed behind the cutoff unit cells demonstrates the spatial-frequency separation inherent to the rainbow trapping effect. Furthermore, the wave energy concentration characteristic of rainbow trapping is also verified, as evidenced by significantly enhanced stress at the gaps where flexural wave propagation stops. The spatial-frequency separation and energy concentration of the SGMB provide multiple enhanced bands for high-frequency energy harvesting. Moreover, this prevents the encounter of the two flexural waves introduced from dual sources, thereby avoiding the corruption of information in the electrical signals.

### 3.2. Piezoelectric Output Simulation

**Figure 8:**
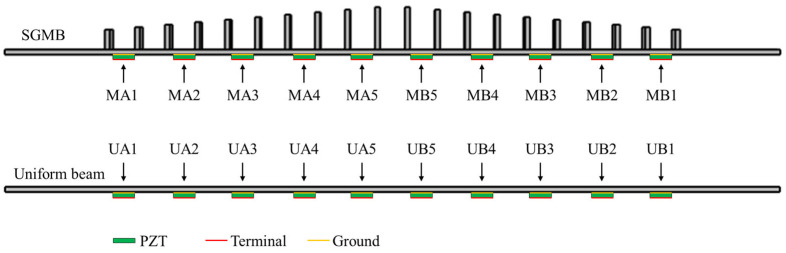
The unit for evaluating the output performance.

Based on the analysis results of the aforementioned frequency domain simulation, a study on piezoelectric energy harvesting is conducted by utilizing the wave energy concentration of the rainbow trapping phenomenon. As shown in Figure 8, the PZTs are attached to the bottoms of the pillar gaps, with each such patch constituting a piezoelectric unit cell. This configuration enables quantitative evaluation of the energy harvesting performance of the gradient beam structure compared to the uniform beam, and its response to different incident frequencies. A coupled solid mechanics-piezoelectric multiphysics simulation was performed using COMSOL Multiphysics. The piezoelectric material was set as PZT-5H from the built-in material library, with dimensions set to 10 × 10 × 0.2 mm. The mechanical damping of the piezoelectric material was represented by an isotropic loss factor, which was set to $\eta_{s}=0.001$. The voltage across its two electrodes corresponds to the open circuit output voltage of the PZT. MAn and MBn denote the nth PZT attached on the symmetrically opposite sides of the metamaterial structure. Similarly, UAn and UBn represent the PZTs on the uniform beam at positions corresponding to those in the SGMB. Due to the symmetrical design of SGMB, only the piezoelectric output of Set MA is simulated and analyzed, as the right-side structure exhibits identical characteristics. Therefore, a vibration source for generating flexural waves is set at the left end of the model, with the acceleration specified as $1\;m/s^{2}$ and the cutoff frequency used as the sweep frequency for this analysis. Meanwhile, the right end of the model was set as a fixed boundary condition. To achieve more accurate computating results for the piezoelectric output simulation, the pre-defined extra fine global element size was employed in the mesh configuration.

**Figure 9:**
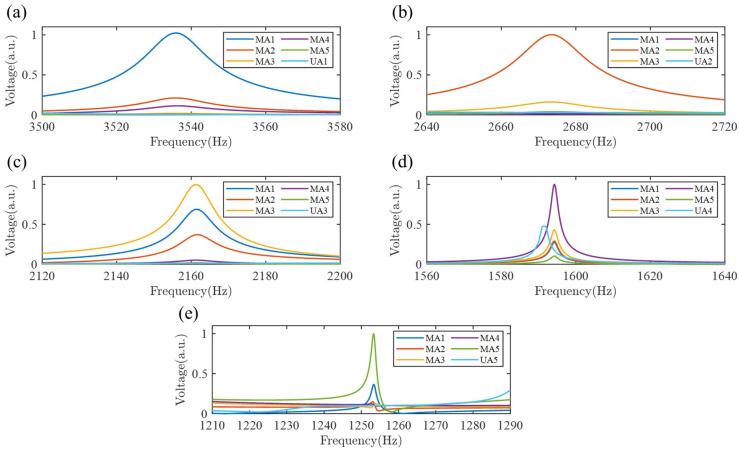
The output voltages of PZTs in the cutoff frequency bands: (**a**) unit MA1, (**b**) unit MA2, (**c**) unit MA3, (**d**) unit MA4 and (**e**) unit MA5.

Figure 9 presents the voltage response of units MA1 to MA5 in the SGMB relative to the uniform beam, across a range of excitation frequencies. The figure displays five frequency bands arranged from high to low: 3500–3580 Hz, 2640–2720 Hz, 2120–2200 Hz, 1560–1640 Hz, and 1210–1290 Hz. These bands correspond to the cutoff frequencies of MA1 to MA5 in the SGMB, respectively, and are described as the cutoff frequency bands. Based on the wave energy concentration effect, the MA1 to MA5 patches all demonstrate excellent energy harvesting capabilities within their respective frequency bands. Taking the MA1 as an example, when the flexural wave frequency is between 3500 and 3580 Hz, the wave is reflected at the location of second pillar. The superposition of the incident and reflected waves causes energy to accumulate at MA1. As a result, the PZT here experiences maximum stress and deformation, leading to the highest output voltage. Since the flexural wave attenuates rapidly after propagating to the MA1 unit cell, the output voltages of the subsequent four PZTs remain minimal, while the output voltage UA1 from the PZT at the corresponding position on the uniform beam is also very weak due to the absence of the rainbow trapping effect.

## 4. Experimental Verification

### 4.1. Experimental Calibration

**Figure 10:**
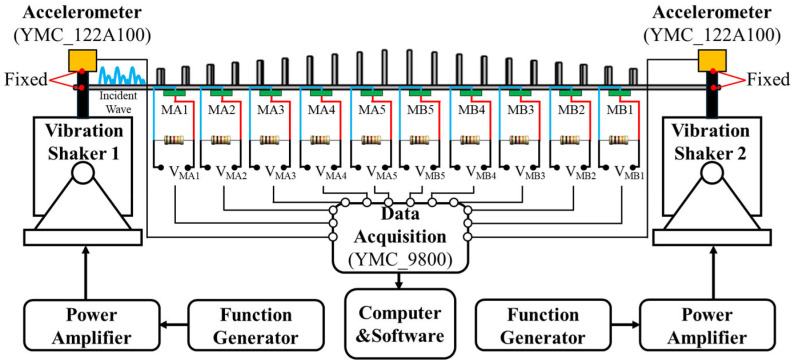
Experimental setup for frequency response.

For a more comprehensive evaluation of the frequency domain response in the designed SGMB, a prototype was fabricated and experimentally tested via a vibrational exciter system. As presented in Figure 10, the SGMB prototype was fixed to a shaker, and its response was evaluated through a frequency sweep test. Consistent with the simulation setup, ten PZTs measuring 10 × 10 × 0.2 mm were bonded at the base of the gaps at locations MA1–MA5 and MB1–MB5. When excited by the shaker, the PZT generates electric charges on its upper and lower surfaces, which power the resistor and create an output voltage signal. Guided by the analysis in Section 2.3, to ensure stable voltage measurement, parallel resistors of 1 MΩ are employed. This resistance considerably exceeds the 40 kΩ benchmark. Additionally, two acceleration sensors (YMC 122A100 IEPE, sensitivity: $9.84\;mV/ms^{2}$, YMC Piezotronics Inc., Yangzhou, China) were installed at both ends of the SGMB structure to capture the input signals from the shakers. The frequency response of the SGMB under a single vibration source is first analyzed. The function generator on the left drives Shaker 1 (YMC Piezotronics Inc.) to produce vibration signals of various frequencies, while Shaker 2 on the right remains inactive and only serves to fix the SGMB, thereby forming a fixed-fixed beam. The response signals of the MA1–MA5 and MB1–MB5 PZTs were then recorded through the data acquisition system for frequency response evaluation.

**Figure 11:**
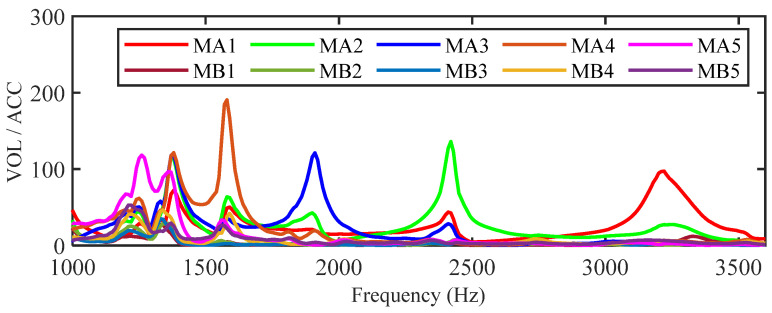
Frequency response of the SGMB with single vibration source.

Displayed in Figure 11 are the frequency sweep results, with the normalized ratio of output voltage (VOL) to input acceleration (ACC) from the left shaker plotted against frequency. Multiple enhanced frequency bands are observed within the range of 1000–3500 Hz, resulting in distinct output voltage peaks for the MA1–MA5 PZTs. This also indicates the wave energy concentration at the corresponding unit cell for these frequencies. Although the MB1–MB5 unit cells are designed with the same cutoff frequencies, the wave energy is predominantly concentrated and harvested by the left-side gradient structure due to the left-side excitation. Consequently, only attenuated flexural waves propagate to the right-side gradient structure, resulting in very low output voltages from the MB1–MB5.

Additionally, Table 3 elucidates the cutoff frequencies of the MA1–MA5 in the SGMB prototype as well as the finite element predictions. A strong agreement is evident between the experimental frequency response of the SGMB and the predictions of the finite element model presented in Figure 7. These findings confirm the effective performance of the SGMB prototype in spatial-frequency separation and wave energy concentration as designed.

To further analyze the frequency response of the SGMB prototype under simultaneous excitation from both the left and right vibration sources, a comparative experiment was conducted using the test platform shown in Figure 10. Two function generators were used to drive Shaker 1 and Shaker 2 (Sinocera Piezotronics Inc., Yangzhou, China), respectively, simultaneously producing vibration signals with various frequencies. It is worth noting that both shakers were driven by independent random signals, with no synchronization in their starting time or sweeping speed. Neither phase alignment nor anti-phase condition was deliberately introduced during the test.

**Figure 12:**
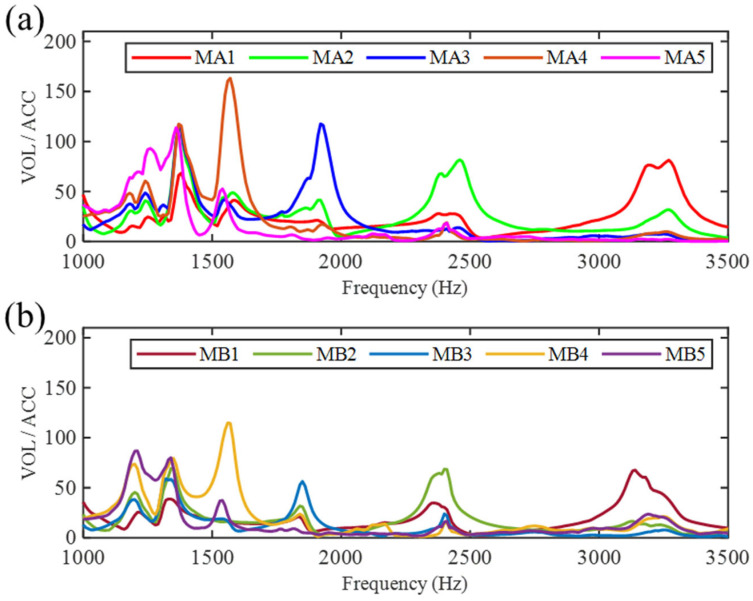
Frequency response of the SGMB with dual vibration source: (**a**) frequency response of MA1–MA5 PZTs and (**b**) frequency response of MB1–MB5 PZTs.

Figure 12 shows the result of the comparative test. Figure 12a presents the frequency responses of the MA1–MA5, where the voltage is normalized by the acceleration of Shaker 1. It can be observed that these test results are similar to those in Figure 11, with five enhanced frequency bands within the range of 1000–3500 Hz. These bands, respectively, amplify the vibration signals and output voltages at the five corresponding unit cell. Similarly, Figure 12b displays the frequency responses of the MB1–MB5, which are normalized by the acceleration of Shaker 2. Compared with the result in Figure 11, the excitation provided by Shaker 2 enables the MB1–MB5 to exhibit identical cutoff frequency bands and similar signal enhancement. Based on the frequency response results, the SGMB prototype can simultaneously process signals from two different vibration sources. By providing multiple discrete frequency bands within 1000–3500 Hz, it greatly improves the efficiency of vibration energy harvesting and significantly attenuates the destructive interference of flexural waves. This capability facilitates powering the self-sensing devices in high-frequency environments for condition monitoring, while avoiding the attenuation and corruption of electrical signals.

### 4.2. Performance Under Vibration Excitation

**Figure 13:**
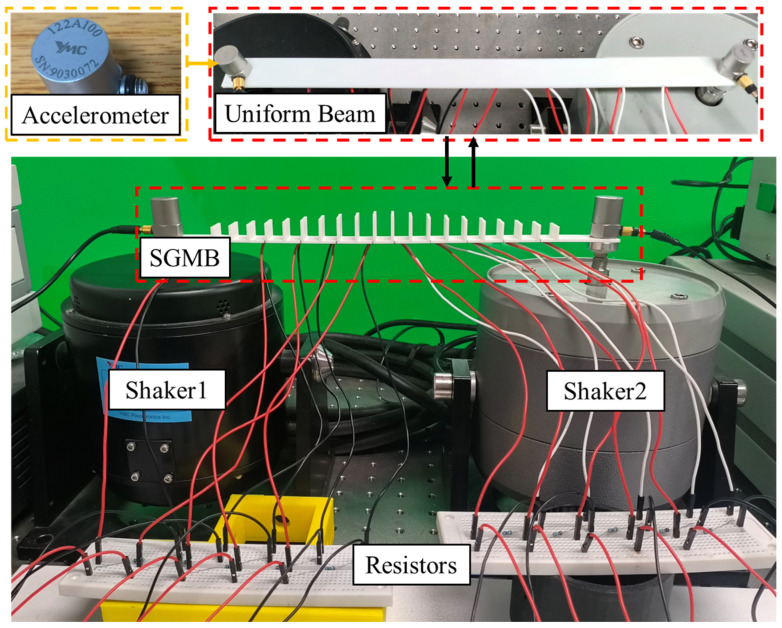
Experimental setup for energy harvesting.

To validate the high-frequency energy concentration and harvesting performance of the proposed SGMB, energy harvesting experiments were conducted on a shaker platform. The shaker simulated linear reciprocating vibration to evaluate the output performance of SGMB under base excitation. Figure 13 presents the SGMB prototype and the uniform beam used for the energy harvesting experiment, as well as the test platform setup.

**Figure 14:**
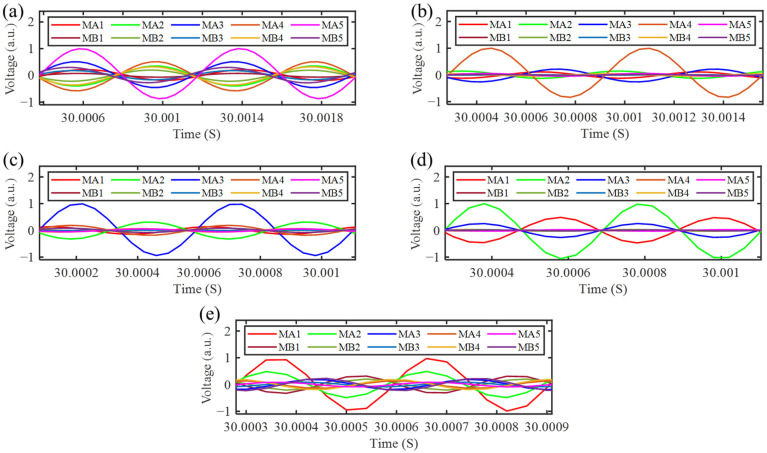
Output performance at distinct frequencies: (**a**) 1250 Hz, (**b**) 1570 Hz, (**c**) 1950 Hz, (**d**) 2410 Hz and (**e**) 3210 Hz.

Energy harvesting tests were conducted on this platform to assess the energy harvesting performance of the SGMB prototype under vibrational excitation. The energy harvesting performance under a single vibration source was analyzed. Shaker 1 on the left was driven to generate vibration signals at 3210 Hz, 2410 Hz, 1950 Hz, 1570 Hz, and 1250 Hz, corresponding to the cutoff frequencies of MA1–MA5, respectively. Shaker 2 on the right remained inactive and served only as a fixture for the SGMB. It is worth noting that since the evaluation focuses on the energy output performance, the resistors with resistance value in 4 –13 kΩ should be selected according to the conclusions in Section 2.3. Therefore, the 10 kΩ resistance was chosen to ensure that all five PZTs achieve high output power at their corresponding cutoff frequencies. Under vibrational excitations at different cutoff frequencies, the voltage signals generated by the ten PZTs were recorded for one minute using a data acquisition card with a sampling rate set to 25 kHz. Figure 14 shows the time domain waveform analysis of the ten PZTs on the SGMB at the five cutoff frequencies. When excited at their respective cutoff frequencies, the corresponding MA5 to MA1 exhibits the maximum output voltage amplitude, indicating that most of the flexural wave energy is converged at these unit cells. Consequently, the output voltage amplitudes of the MB1–MB5 on the right side remain minimal.

**Figure 15:**
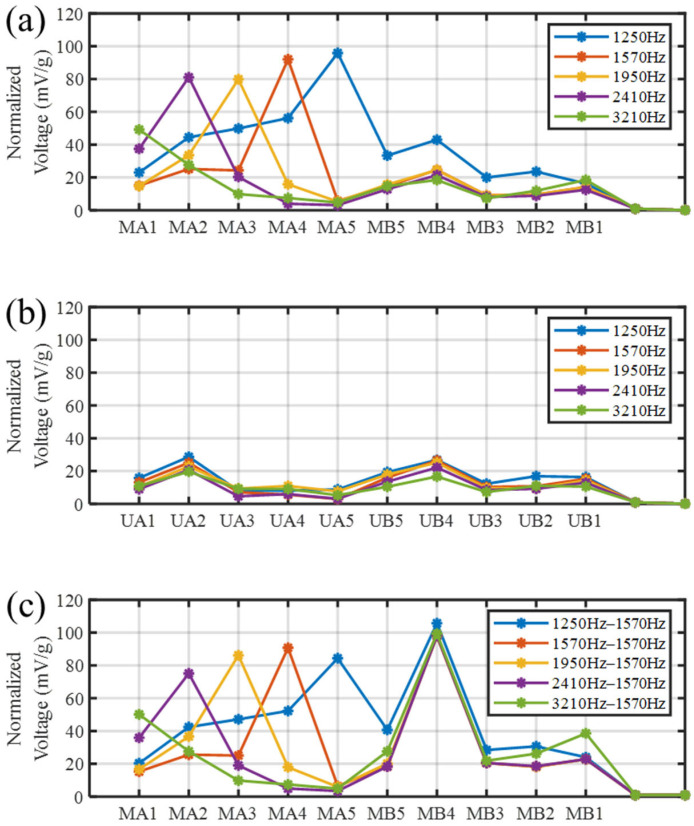
Comparison of Normalized Output Voltage: (**a**) single-side excitation on SGMB, (**b**) single-side excitation on uniform beam and (**c**) double-side excitation on SGMB.

Energy harvesting was performed on the uniform beam using the test platform shown in Figure 13 to analyze its performance under single-side excitation. The experimental result was compared with those of the SGMB. Figure 15a,b illustrate the output voltages of the SGMB and the uniform beam under single-side cutoff frequency excitation. The voltage values represent the RMS values of the AC voltage, these values are normalized by the input acceleration, defining the voltage sensitivity. At their corresponding cutoff frequencies, the MA1–MA5 exhibit considerable voltage sensitivity, with the lowest MA1 reaching 49 mV/g and the highest MA5 achieving 96 mV/g.

In contrast, due to the absence of enhancement from the rainbow trapping effect, the voltage sensitivity of the UA1–UA5 on the uniform beam remains very weak, not exceeding 29 mV/g at maximum, with no clear correlation between excitation frequency and PZT location. The experimental results verify the enhancement of the SGMB on the energy harvesting performance, as well as the correspondence between the cutoff frequencies and spatial locations. It is worth noting that the voltage sensitivity of MB1–MB5 is very close to that of UB1–UB5, demonstrating that the flexural wave energy input from the left side is captured by MA1–MA5, leaving the right-side gradient structure unable to exhibit the rainbow trapping effect.

To demonstrate the energy harvesting capability of the introduced SGMB under simultaneous dual vibration sources, a test was performed on the same platform shown in Figure 13. The energy harvesting performance under double-side excitation was analyzed and compared with the results obtained from single-side excitation. During the experiment, the vibration frequency of Shaker 2 was maintained at 1570 Hz, while the vibration frequency of Shaker 1 was set to the five cutoff frequencies, respectively. Operating at a 25 kHz sampling rate, the data acquisition card recorded for one minute under each frequency condition. Figure 15c shows the voltage sensitivity of each PZTs in the SGMB under dual vibration excitations. Compared with the single-side excitation results, the voltage sensitivity of MA1–MA5 remains largely consistent. Since 1570 Hz is the cutoff frequency of the MB4, the flexural wave energy input from the right side is captured by MB4. This process remains unaffected by frequency variations on the left side. The experimental results demonstrate that the SGMB energy harvester can simultaneously harvest energy from high-frequency flexural waves energy from different vibration sources. The gradient structures on both the left and right sides effectively exhibit the spatial-frequency separation and wave energy concentration of rainbow trapping, operating without mutual interference. This characteristic facilitates high-frequency broadband dual-source energy harvesting, thereby enhancing the power supply for self-sensing devices.

### 4.3. Performance on Rotor Test Rig

**Figure 16:**
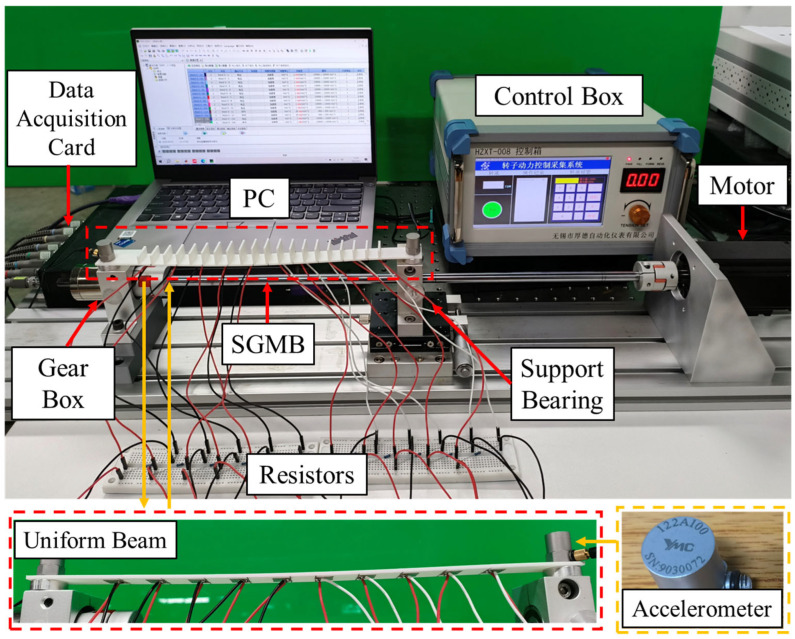
Energy harvesting on rotor test rig.

Harvesting piezoelectric vibration energy to power wireless self-sensing devices in condition monitoring is challenging, as harvesters must operate in complex and broadband environments. To evaluate the reliability and output performance of the proposed SGMB under such realistic conditions, a test was performed on a rotor test rig. As shown in Figure 16, the SGMB is mounted on the rotor test rig, with its left end fixed to the gearbox base and its right end secured to the bearing base. The vibrational energy generated by these two rotating components during operation is converted into electrical energy and harvested by the MA1–MA5 and MB1–MB5 piezoelectric patches. Meanwhile, two accelerometers were fixed at the left and right ends, to record the input vibration signals. Sampling was performed at 25 kHz using the data acquisition card. The motor speed was set to a low speed of 300 rpm and a high speed of 3300 rpm, with data sampled for one minute at each speed to comprehensively evaluate the energy harvesting performance of the SGMB under different rotational speeds. Following the completion of the above experiments, the uniform beam was installed on the rotor test rig to repeat the energy harvesting tests, and the results were compared with that from the SGMB.

**Figure 17:**
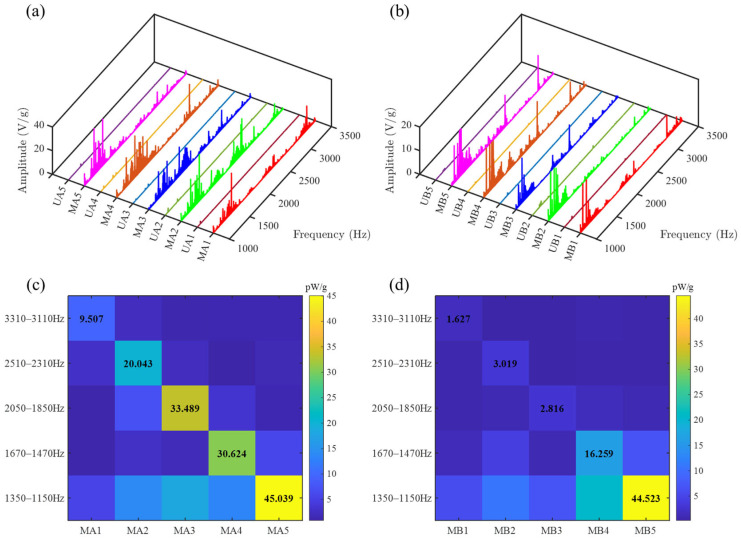
Output performance on test rig under 300 rpm: (**a**) comparison of gearbox vibration spectra, (**b**) comparison of bearing vibration spectra, (**c**) energy concentration of gearbox vibration signals on SGMB and (**d**) energy concentration of bearing vibration signals on SGMB.

**Figure 18:**
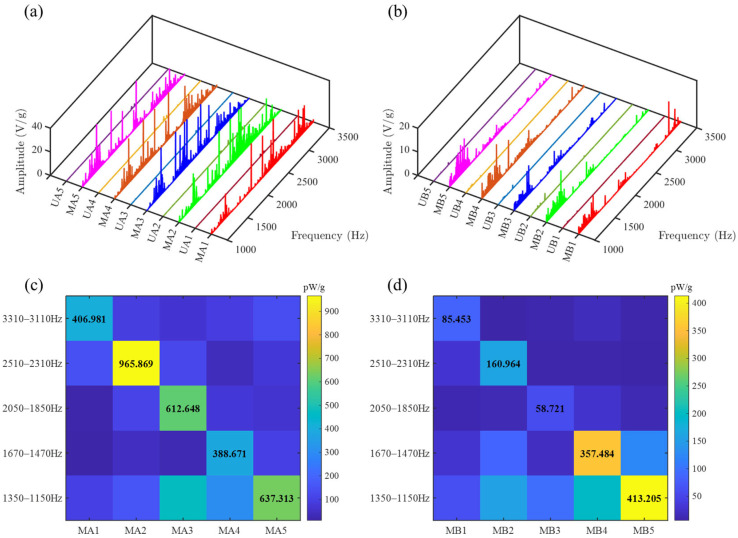
Output performance on test rig under 3300 rpm: (**a**) comparison of gearbox vibration spectra, (**b**) comparison of bearing vibration spectra, (**c**) energy concentration of gearbox vibration signals on SGMB and (**d**) energy concentration of bearing vibration signals on SGMB.

The output voltage of each PZT was processed through Fourier transform and normalized by the acceleration at each frequency, yielding the spectral comparison diagrams shown in Figure 17a,b and Figure 18a,b. Across the common operating speed range, the amplitudes of the gear vibration signal (collected by MA1–MA5) and bearing vibration signal (collected by MB1–MB5) enhanced by the SGMB significantly surpass those collected by the uniform beam (UA1–UA5 and UB1–UB5). Furthermore, due to the higher intensity of gear vibration compared to bearing vibration, the energy density of MA1–MA5 is substantially greater than that of MB1–MB5 at high rotational speed.

The frequency bands formed by utilizing the cutoff frequencies as center frequencies, with upper and lower sideband widths of 100 Hz, are defined as cutoff frequency bands. It can be observed that within the cutoff frequency bands, the corresponding PZTs exhibit significant energy concentration and high output voltages. The voltage value of each PZT was averaged across its cutoff frequency band. The output power of each PZT within its cutoff frequency band is computed based on the averaged voltage and resistance value and normalized by the acceleration, yielding the results shown in Table 4. Due to the energy concentration effect in the symmetrical gradient metamaterial structure, the wave energy from the two sources is concentrated at their corresponding unit cells. This effectively enhances the energy harvesting efficiency and prevents energy dissipation caused by destructive interference. As a result, the metamaterial beam exhibits a marked advantage in normalized power compared to the uniform beam. The output power advantage of the metamaterial beam becomes more pronounced at the high rotation speed as a result of enhanced vibration. Compared to uniform beam, the SGMB improves the energy harvesting efficiency by a factor over 100 within the cutoff frequency bands.

A comparison of the normalized power generated by PZTs on the SGMB in each cutoff frequency is presented in Figure 17c,d and Figure 18c,d. The symmetrical PZTs consistently exhibit the highest output power in their respective cutoff frequency band, which further confirms the capability of SGMB for multi-band enhancement and dual-source energy harvesting. The experimental results demonstrate that the SGMB energy harvester can simultaneously collect energy from dual different vibration sources, including signals from gears and bearings. By leveraging the spatial-frequency separation and wave energy concentration of rainbow trapping, the high-frequency broadband energy harvesting from dual sources is achieved.

## 5. Conclusions

Conventional piezoelectric energy harvesters often disregard high-frequency energy, which is weak but contains abundant condition-monitoring information. Furthermore, destructive interference between multiple vibration sources results in attenuation and corruption of high-frequency signals. To address this issue for powering self-sensing devices, an innovative vibration energy harvester was designed based on a symmetrical metamaterial structure integrated with multiple piezoelectric patches. The design and validation were informed by numerical and experimental evaluation of its power generation performance. The conclusions can be summarized as follows:

(1) Structural design and optimization were conducted using a COMSOL model. The results show that the progressively increased height of the rectangular pillars yields multiple enhancement bands in high-frequency range.
(2) The electrical output of the energy harvesting unit can be modeled as a single degree of freedom (SDOF) system, allowing for accurate determination of the optimal resistance for the energy harvesting circuit.
(3) The proposed SGMB exhibits multiple discrete cutoff bands within the frequency range of 1000 Hz to 3500 Hz. Compared to a uniform beam, the metamaterial beam demonstrates an overwhelming advantage in electrical power generation across these cutoff bands.
(4) The symmetrical gradient structure effectively mitigates the destructive interference of flexural waves from two vibration sources, thereby facilitating broadband dual-source energy harvesting while avoiding the corruption of electrical signals.

Furthermore, the proposed energy harvester can be extended to a self-sensing system by processing the voltage signals converted from PZTs. Nevertheless, the mutual interference between multiple signals and the enhancement of fault signals within the cutoff bands require further optimization and exploration in future research.

## Figures and Tables

**Table 1:** Key parameters of PZT patches [35].

Item	Dimensions mm	$\mathrm{Density}\;\rho_{P}(kg/m^{3})$	Stiffness $\;E_{P}(GPa)$	Capacitance $\;C_{P}(nF)$	Electromechanical Factor α
Value	10 × 10 × 0.2	7500	66	12	0.68

**Table 2:** The height and cutoff frequency of rectangular pillars.

Number	$H_{n}/mm$	Gap Name	Cutoff Frequency by FEA (Hz)
1	8		3974
2	9	MA1	3379
3	10		2896
4	11	MA2	2504
5	12		2184
6	13	MA3	1920
7	14		1685
8	15	MA4	1516
9	16		1359
10	17	MA5	1226

**Table 3:** The cutoff frequency in SGMB prototype.

PZT Name	Cutoff Frequency by FEA (Hz)	Cutoff Frequency by Prototype (Hz)
MA1	3379	3210
MA2	2504	2410
MA3	1920	1950
MA4	1516	1570
MA5	1226	1250

**Table 4:** Output power comparison of SGMB and uniform beam.

PZT Name	Power (pW/g) at 300 rpm	Power (pW/g) at 3300 rpm	PZT Name	Power (pW/g) at 300 rpm	Power (pW/g) at 3300 rpm
MA1	9.507	406.981	UA1	0.025	1.930
MA2	20.043	965.869	UA2	0.329	19.687
MA3	33.489	612.648	UA3	0.014	0.282
MA4	30.624	388.671	UA4	0.309	5.531
MA5	45.039	637.313	UA5	0.181	2.218
MB5	44.523	413.205	UB5	0.129	1.315
MB4	16.259	357.484	UB4	0.083	0.847
MB3	2.816	58.721	UB3	0.028	0.598
MB2	3.019	160.964	UB2	0.121	2.940
MB1	1.627	85.453	UB1	0.014	0.271

## Data Availability

The data presented in this study is available upon request from the corresponding authors.
